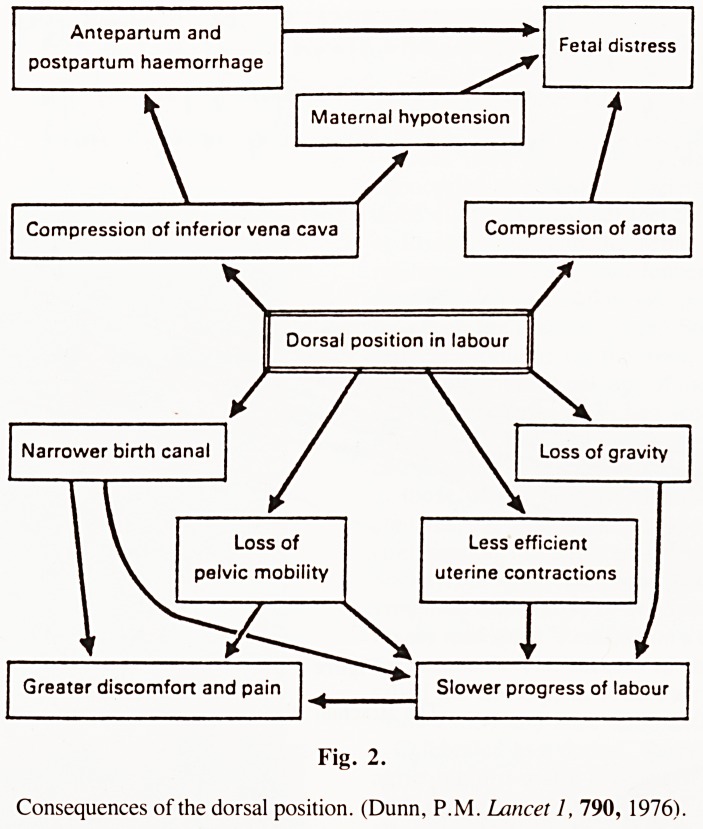# Childbirth, Lessons from the past

**Published:** 1991-12

**Authors:** Peter M. Dunn


### "Childbirth: Lessons from the past"

**Peter M. Dunn, MD, FRCP, FRCOG**

The late Sir Robert Hutchison used the following words as he gave his Harveian oration in 1931. "Look round this room in which we meet. Is it not a noble library indeed, but is it not also a mausoleaum? And how many facts which men are at present hunting for, and theories which are even now being put forward as new, lie already buried in these shelves?" This observation is particularly apposite to childbirth and the perinatal period because, following the introduction of aseptic surgery and anaesthesia at the end of the last century, the physician accoucheurs who were also early paediatricians became almost overnight, surgeon gynaecologists and a great deal of obstetric and neonatal knowledge sank into oblivian during the next half century, being "discovered" again in the last thirty years. Among the many, many examples that might be given are:

Asphyxiation of newborn infants as a result of tight umbilical binders was first described by William Smellie in 1752 but reported afresh by Professor John Emery in 1965.

There was the re-discovery in the 1970s using ultrasound that the fetus breathed in utero, after thirty years of denial by scientists following the work of Sir Joseph Barcroft. Yet this fact was well established in the 19th century. John Ballantyne wrote in 1902 — "Nature makes no leaps, but prepares beforehand for the transitions of life and even for those of them that seem at first sight so abrupt as does the establishement of pulmonary respiration in the place to placental. She makes the necessary transitions easy. Truly, birth marks not a beginning but a stage in life's journey." Ballantyne it was too who introduced antenatal care and foreshadowed the modern concept of clinical genetics and the pre-conception clinic in his writings on "germinal therapeutics".

The ventouse suction method of delivery was introduced into modern obstetrics in Scandinavia in the 1950s. Less well known is the fact that the technique was first used by Arnot in this country in 1829 and by James Young Simpson in the 1840s.

The Drew-Smythe catheter, described by Bristol's first professor of obstetrics and gynaecology, was very similar to an instrument used by a Scottish obstetrician in the 18th century and medical methods of inducing labour using oxytocic agents were well described by Tanner and others in the 19th century.

In 1961 Nelligan described the use of elastic bands to occlude the umbilical cords of newborn babies. Yet this technique had been described and used by Pierre Budin of Paris in the 1870s.

The intravenous infusion of glucose and bicarbonate for babies with respiratory distress syndrome was described by Robert Usher of Montreal in the early 1960s. Yet Schückring of Berlin was infusing fructosate of soda through the umbilical vein of newborn babies with breathing problems as early as the turn of the century.

Many people think that artificial ventilation of newborn babies was pioneered by Paul Swyer in Toronto in the early 1960s, yet in spite of the technical difficulties, a negative pressure neonatal ventilator had been produced in France before the end of the last century. Likewise many credit George Gregory and his colleagues in San Francisco with the introduction of continuous positive airway pressure in the treatment of respiratory distress syndrome in 1971. Yet the fact remains that this technique was being used in Germany as early as 1909.

Perhaps the most significant lesson from the past still remains to be fully appreciated by modern practice. Historical research reveals that until the 17th century women throughout the world had from the most ancient times laboured and delivered in an upright position either standing, kneeling, sitting, crouching or squatting. It was only after the entry of man-midwives (who later became physician accoucheurs, obstetricians and finally gynaecologists) that women were expected to assume the dorsal position in bed for childbirth. As an American anthropologist,

**Fig. 1.**

Delivery in Africa (after Felkin, 19th c.)

Richard Atwood, wrote in 1976 — "The field of Western obstetrics may be considered a culture because the learned behaviour patterns become accepted practice and universally used and then passed on to the next generation . . . The horizontal delivery positions are orientated towards making it easier for the obstetrician to do his job, although the patient may not always be comfortable . . . as long as the field of obstetrics remains orientated towards clinical obstetrical problems, the patient will continue to be treated like a pathological case and the delivery will continue to be considered as an operation. . . If the mother chooses to be delivered in a hospital by an obstetrician (in USA) her delivery position choices are usually limited to just one, the lithotomy position on a delivery table with leg supports . . .". Yet even to-day throughout those parts of the developing world which have not yet been permeated by Western obstetrics, women still retain an upright position for labour and delivery. Research in the last 15 years has revealed the advantages of an upright posture and the mobility that goes with it and the disadvantages of a static, recumbent posture (Fig. 2). As I wrote in 1978: "A great deal of obstetric effort has lately been directed towards trying to make labour shorter, safer and less painful. Let us hope that more attention will now be paid to maternal posture and mobility. Then augmented labour and maternal analgesia with their unwanted side effects and hazards might be needed less often."

**Fig. 2.**

Consequences of the dorsal position. (Dunn, P.M. *Lancet 1,* **790,** 1976).

## Figures and Tables

**Fig. 1. f1:**
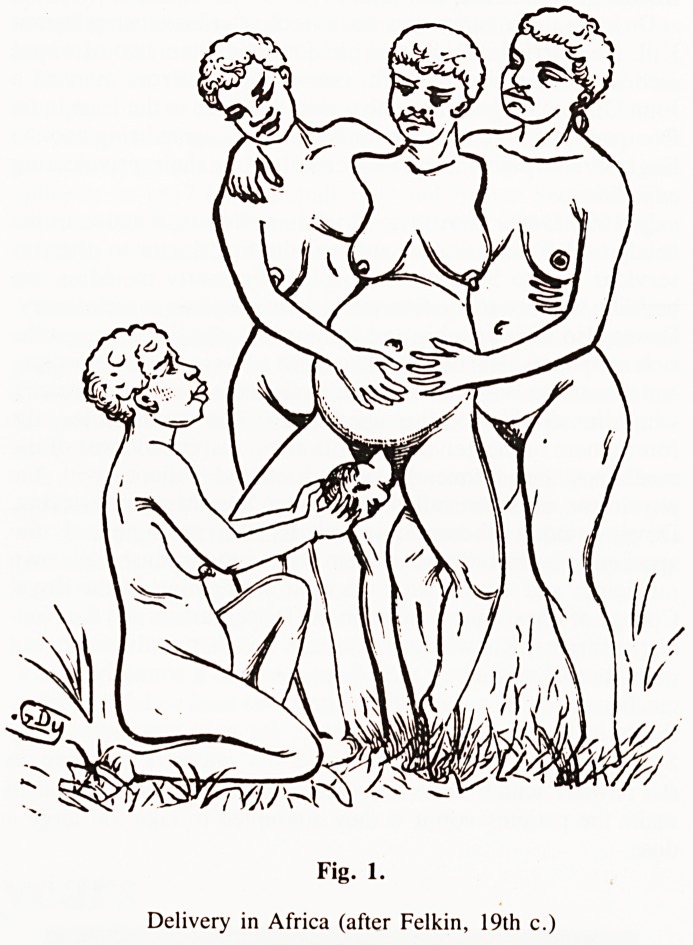


**Fig. 2. f2:**